# Near-Fatal Hemodynamic Collapse during Recovery from Staged Bladder Tumor Resection in a Patient with Giant Left Atrial Myxoma: A Case for Immediate Sequential Surgery

**DOI:** 10.70352/scrj.cr.25-0832

**Published:** 2026-03-20

**Authors:** Kazuko Tokiya, Michiyoshi Sanuki, Shigeaki Kurita

**Affiliations:** Department of Anesthesiology, Critical Care and Pain Medicine, NHO Kure Medical Center and Chugoku Cancer Center, Kure, Hiroshima, Japan

**Keywords:** left atrial myxoma, bladder carcinoma, staged surgery, immediate sequential surgery, cardiopulmonary bypass, noninvasive positive pressure ventilation, case report

## Abstract

**INTRODUCTION:**

Surgical management of a large, obstructive left atrial (LA) myxoma coexisting with an active bleeding source presents a significant clinical dilemma. The systemic heparinization required for cardiopulmonary bypass (CPB) carries a high risk of exacerbating hemorrhage, whereas delaying cardiac surgery leaves the patient vulnerable to fatal myxoma-related complications.

**CASE PRESENTATION:**

A 77-year-old woman presented with gross hematuria from an 8-cm bladder tumor. Preoperative evaluation revealed a giant (74 × 40 mm) LA myxoma prolapsing through the mitral valve, causing functional mitral stenosis. A multidisciplinary team elected for a “urology-first” staged approach. Transurethral resection of the bladder tumor (TURBT) was performed under general anesthesia with successful hemostasis. However, 30 min after extubation, the patient developed acute respiratory failure and cardiogenic shock due to myxoma incarceration. She was stabilized with noninvasive positive pressure ventilation and fluid management before being transferred to the ICU. Emergency myxoma resection was performed 18 h later. No significant bladder bleeding occurred despite full heparinization.

**CONCLUSIONS:**

This case demonstrates that the post-anesthesia recovery period is a window of extreme vulnerability for patients with obstructive myxomas, likely due to emergence-related tachycardia. The “urology-first” staged approach, while logical for bleeding control, failed to prevent a life-threatening cardiac event. Accumulating evidence suggests that bleeding risk following adequate hemostasis at TURBT is low, even under anticoagulation. For patients with high-risk cardiac lesions, an immediate sequential surgical strategy—proceeding directly to cardiac surgery under the same anesthesia once non-cardiac hemostasis is achieved—should be strongly considered to minimize the duration of risk.

## Abbreviations

CPB
: cardiopulmonary bypass
LA
: left atrial
NPPV
: noninvasive positive pressure ventilation
TAE
: transcatheter arterial embolization
TEE
: transesophageal echocardiography
TURBT
: transurethral resection of the bladder tumor
VA-ECMO
: veno-arterial extracorporeal membrane oxygenation

## INTRODUCTION

Primary cardiac tumors are rare, with myxoma being the most frequent benign subtype in adults.^1)^ Despite their benign histology, LA myxomas can be functionally malignant, causing valvular obstruction, embolization, and sudden death.^2)^ The standard of care is prompt surgical excision under CPB.^3)^

Management becomes complex when an LA myxoma coexists with a condition requiring urgent non-cardiac surgery, particularly one with active bleeding. This creates a therapeutic dilemma: systemic heparinization (typically 300–400 U/kg) for CPB may precipitate catastrophic hemorrhage from the non-cardiac site,^4)^ while delaying cardiac surgery exposes the patient to myxoma-related complications exacerbated by perioperative stress.^5)^

Evidence guiding surgical sequencing in such cases is limited.^4–7)^ We present a case of a 77-year-old woman with a giant, obstructive LA myxoma and a concurrent hemorrhagic bladder tumor. We describe a “urology-first” staged strategy that, although it achieved intraoperative stability, resulted in near-fatal hemodynamic collapse during the recovery phase. We discuss the mechanisms of this failure and advocate for an “immediate sequential” surgical strategy.

## CASE PRESENTATION

A 77-year-old woman (height 153 cm, weight 47 kg) presented with persistent gross hematuria and anemia (hemoglobin 9.1 g/dL). Contrast-enhanced CT revealed an 8-cm intravesical mass with contrast extravasation. Incidentally, the chest CT showed a large 50-mm filling defect in the left atrium.

**Fig. 1:**
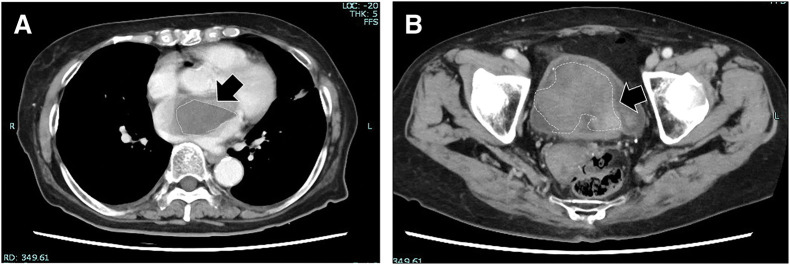
Preoperative imaging (**A**) Chest CT showing a 50-mm left atrial filling defect (arrow). (**B**) Pelvic CT showing an 8-cm hemorrhagic bladder tumor (arrowhead).

Transthoracic and TEE demonstrated a mobile, pedunculated mass (74 × 40 mm) attached to the fossa ovalis. The mass prolapsed across the mitral valve during diastole, causing functional mitral stenosis (mean gradient 13.7 mmHg) (**Fig. 1**). Left ventricular systolic function was preserved.

Surgical Strategy: A multidisciplinary team (cardiac surgery, urology, and anesthesiology) discussed the following three options:

1. Cardiac-first: Rejected due to the risk of uncontrollable bladder hemorrhage during heparinization.
2. Simultaneous Combined: Rejected for similar bleeding concerns.
3. Urology-first Staged: Selected to prioritize hemostasis via TURBT, followed by cardiac surgery at a later date.

**Fig. 2:**
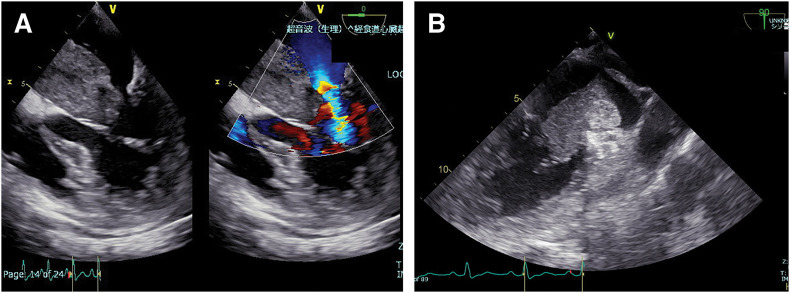
Transesophageal Echocardiography (**A**) Mobile LA mass prolapsing into the mitral orifice. (**B**) Imminent ball-valve obstruction during transient fluid reduction. LA, left atrial

The Procedure and Subsequent Course: TURBT was performed under general anesthesia with continuous TEE monitoring.^8,9)^ Before anesthesia induction, the cardiac surgery team inserted bilateral femoral arterial and venous sheaths to enable rapid establishment of CPB or VA-ECMO should acute hemodynamic deterioration occur.^10–12)^ A primed CPB circuit with an oxygenator was kept on standby in the operating room throughout the procedure. Anesthesia was maintained with propofol and remifentanil. The urologists performed piecemeal resection using a transurethral resection in a saline system. The patient remained hemodynamically stable throughout the 70-min procedure. A transient episode of impending ball-valve obstruction was noted on TEE when irrigation flow was reduced; however, this resolved with fluid loading (**Fig. 2**).

The patient was extubated and transferred to the ward. However, 30 min later, she developed acute decompensation with severe dyspnea, sinus tachycardia (130 bpm), and hypotension (80/50 mmHg). Arterial blood gas showed severe hypercapnic respiratory failure (pH 7.05 and PaCO_2_ 82.4 mmHg). Bedside rescue TEE confirmed that the mass was wedged at the mitral annulus, causing near-total inflow obstruction consistent with acute myxoma incarceration.^13–15)^

The patient was managed with bilevel positive airway pressure and transferred to the ICU. Simultaneously, cautious fluid resuscitation and diuretics were initiated. Over the following hours, hemodynamics gradually stabilized, with the heart rate maintained at 90 bpm and the mean arterial pressure sustained above 65 mmHg. This was believed to be due to the positive pressure from NPPV reducing right ventricular preload, which, through ventricular interdependence, shifted the interventricular septum and partially alleviated the myxoma incarceration.^16,17)^ The patient’s condition improved sufficiently to enable controlled surgical preparation—including mobilization of the cardiac surgery team, operating room setup, and optimization of coagulation parameters—rather than necessitating a crash-to-bypass scenario. Consequently, the urology and cardiac surgery teams elected to proceed with the operation as a semi-emergency the following morning. Although VA-ECMO via the pre-placed femoral sheaths was considered a bridge to surgery, it was ultimately unnecessary.

Thus, emergency sternotomy was performed 18 h post TURBT. CPB was established with full heparinization (ACT >480 s). The mass was excised en bloc. Crucially, no bladder bleeding occurred during or after cardiac surgery. The patient was discharged on POD 15 without further complications. Pathology confirmed a myxoma and high-grade urothelial carcinoma.

## DISCUSSION

This case illustrates the narrow safety margin in managing large, obstructive LA myxomas with concurrent bleeding malignancies. Although the “urology-first” staged approach was designed to mitigate bleeding risk, it paradoxically exposed the patient to a life-threatening event during the recovery phase.

### Mechanisms of decompensation: the “vulnerable phase”

The patient was stable during anesthesia but collapsed after extubation. Emergence from anesthesia induces sympathetic activation and tachycardia. In mitral stenosis physiology, tachycardia critically shortens diastolic filling time.^18)^ The elevated heart rate (130 bpm) during recovery likely reduced left ventricular filling and increased LA pressure, leading to tumor incarceration. This indicates that the recovery period is potentially more dangerous than the surgery for these patients. The successful use of NPPV in this case highlights its dual benefit: improving oxygenation and favorably altering cardiac hemodynamics by reducing right ventricular preload.^16,19)^

### Post-TURBT bleeding risk: evidence supporting immediate sequential surgery

A critical question in formulating the immediate sequential strategy is whether the TURBT resection bed can withstand systemic heparinization required for CPB. Recent large-scale data help quantify the actual bleeding risk following TURBT. An analysis of 24100 TURBTs from the ACS National Surgical Quality Improvement Program database reported a perioperative blood transfusion rate of only 1.5% and a surgical re-intervention rate of 1.5%.^20)^ Importantly, Regele et al. demonstrated in a cohort of 583 patients that prophylactic subcutaneous heparin administered at anesthesia induction did not increase clinically significant bleeding after TURBT.^21)^ Furthermore, Konishi et al. showed that continuing antiplatelet and/or anticoagulant agents during TURBT did not increase the risk of severe hemorrhage or blood transfusion, although postoperative clot retention rates were higher (21% vs. 5%).^22)^ These data collectively suggest that once the urologist has confirmed adequate endoscopic hemostasis, the cauterized TURBT resection bed is relatively resistant to hemorrhagic complications even under anticoagulation. This evidence provides a strong rationale for our proposed immediate sequential strategy: the risk of clinically significant bleeding from the TURBT site during subsequent heparinization for CPB appears to be substantially lower than the hemodynamic risk of awakening a patient with a large obstructive myxoma.

### Alternative strategies for persistent post-TURBT bleeding

It should be acknowledged that if endoscopic hemostasis during TURBT is incomplete, proceeding directly to cardiac surgery with full heparinization would pose an unacceptable bleeding risk. In such scenarios, TAE of the vesical arteries offers a viable alternative for achieving hemostasis. TAE has demonstrated immediate bleeding control in approximately 87%–90% of cases of intractable bladder hemorrhage.^23,24)^ If TURBT achieves only partial hemostasis, selective vesical artery embolization could be performed as an interim measure—potentially under the same anesthesia—before proceeding to cardiac surgery. This approach preserves the option of the immediate sequential strategy while addressing the bleeding concern. Therefore, we recommend that the multidisciplinary surgical team have a pre-established contingency plan, including interventional radiology consultation, should post-TURBT bleeding prove refractory to endoscopic management.

### A case for “immediate sequential surgery”

Previous reports have debated the merits of simultaneous versus staged strategies.^4–7,25)^ Our case adds a critical perspective: once endoscopic hemostasis is achieved, the bleeding risk during heparinization may be lower than the hemodynamic risk of waking the patient. The absence of bladder bleeding during our patient’s subsequent cardiac surgery (despite full heparinization) supports this. Therefore, we propose that for patients with obstructive myxomas and treatable bleeding sources, an “immediate sequential strategy” is superior. This protocol involves:

1. Non-cardiac procedure first: Achieve hemostasis (e.g., TURBT) under general anesthesia.
2. Maintenance of anesthesia: Once hemostasis is confirmed, do not extubate.
3. Immediate transition: Proceed directly to sternotomy under the same anesthetic.

This eliminates the hemodynamic volatility of emergence and the “unmonitored” risk window. If post-TURBT hemostasis is inadequate, TAE should be considered a bridge before proceeding to cardiac surgery.

## CONCLUSIONS

A large, obstructive LA myxoma poses significant risks during the recovery phase of non-cardiac surgery. A staged approach with an interval of wakefulness can be fatal. We recommend an immediate sequential surgical strategy—proceeding to cardiac surgery under the same anesthesia once non-cardiac hemostasis is secured—to ensure patient safety. Accumulating evidence on the low bleeding risk after TURBT, even under anticoagulation, supports the safety of this approach. Pre-placed vascular sheaths for emergent CPB/VA-ECMO and contingency planning, including transcatheter arterial embolization, are essential components of the perioperative strategy.

## DECLARATIONS

### Availability of data and materials

Data sharing is not applicable to this article, as no datasets were generated or analyzed during the current study.
